# ‘Leave no one behind’: cochlear implantation under regional anaesthesia in a humanitarian setting – a case series

**DOI:** 10.7189/jogh.16.04342

**Published:** 2026-09-11

**Authors:** Isra Aljazeeri, Mohammad F Alzahrani, Roa Talal Halawani, Hazem Ibrahim Shoukry, Ahmad M Aldhafeeri, Ibrahim Shami, Abdulrahman Hagr

**Affiliations:** 1 Department of Otolaryngology-Head and Neck Surgery, College of Medicine, King Saud University, Riyadh, Saudi Arabia.; 2 Department of Research, King Abdullah Ear Specialist Center (KAESC), King Saud University Medical City, King Saud University, Riyadh, Saudi Arabia; 3 Department of Otorhinolaryngology, Aljaber Ophthalmology and Otolaryngology Specialized Hospital, Ahsa Health Cluster, Ministry of Health, Saudi Arabia.; 4 Research Department, Cochlear Implant Specialized Centers, Ministry of Health, Riyadh, Saudi Arabia; 5 College of Medicine, King Saud University, Riyadh, Saudi Arabia; 6 Cochlear Implant Center, Ohud General Hospital, Madinah Health Cluster, Madinah, Saudi Arabia; 7 Anesthesia Department, Ohud General Hospital, Madinah Health Cluster, Madinah, Saudi Arabia; 8 Hafr Albaten Cochlear Implant Center, Hafr Albaten Health Cluster, Hafr Albaten, Saudi Arabia; 9 King Fahad Medical City, Ministry of Health, Riyadh, Saudi Arabia

**Keywords:** case series, otolaryngology, cochlear implantation, regional anaesthesia, humanitarian, early activation

## Abstract

**Background:**

Cochlear implantation (CI) is the standard of care for severe-to-profound hearing loss. While traditionally performed under general anaesthesia (GA), GA poses significant logistical and safety challenges during humanitarian missions in low-resource settings, often excluding patients with complex medical comorbidities. Regional anaesthesia (RA) offers a practical alternative that avoids GA-related risks, reduces operative time, and allows treatment for otherwise ineligible patients, upholding the humanitarian principle of ‘leaving no one behind’. Therefore, the objective of the study is to report the feasibility, safety, and outcomes of cochlear implantation performed under regional anaesthesia during humanitarian missions, with the implementation of early activation protocols.

**Methods:**

This case series includes eight patients (age range 20–66 years) of Palestinian, Somali, and Senegalese nationality who underwent cochlear implantation under regional anaesthesia during humanitarian missions conducted in Jordan (serving Palestinian patients), Kenya (serving Somali patients), and Senegal (serving Senegalese patients) in April and December 2025.

**Results:**

All eight procedures were successfully completed under regional anaesthesia without conversion to general anaesthesia. The diverse case mix included medically complex patients and challenging surgical anatomy. Early activation was achieved in all cases.

**Conclusions:**

Regional anaesthesia enables cochlear implantation in medically complex patients who cannot undergo general anaesthesia – including those with failed intubation or high cardiac risk – while allowing intraoperative or immediate postoperative activation, ensuring no one is left behind in humanitarian missions. Early activation protocols are achievable with logistical efficiency in resource-limited environments.

Cochlear implantation has become the standard of care for patients with severe-to-profound sensorineural hearing loss, offering significant improvements in quality of life, communication abilities, and social integration [1–4]. Traditionally performed under general anaesthesia, cochlear implantation requires longer operative time, anaesthetic support, and postoperative recovery period [5].

The concept of cochlear implantation under regional anaesthesia (RA) with or without conscious sedation has gained increasing attention over the past decade. The advantages of this approach include avoidance of general anaesthesia-related risks – particularly relevant for elderly patients or those with comorbidities – reduced operative time, faster recovery, and the unique opportunity for intraoperative auditory feedback during electrode insertion and device activation [5–8].

A recent meta-analysis by Walters *et al*. (2022) encompassing 18 studies and 262 patients undergoing cochlear implantation under local anaesthesia with sedation reported a pooled success rate of 99.6% (only one patient needed conversion) without conversion to general anaesthesia, pooled mean operative time found to be shorter than general anaesthesia (approximately 22.59 minutes). There were no specific complications directly caused by local anaesthesia. This review also illustrated a trend into lower cost with local anaesthesia [8].

Cochlear implantation in the humanitarian context needs sophisticated organisation [9]. Surgical missions typically operate in low- and middle-income countries where hearing healthcare infrastructure is fragile or non-existent. In these contexts, general anaesthesia (GA) – the traditional standard for cochlear implantation – poses additional burdens.

Patients with complex medical conditions are often excluded from humanitarian cochlear implant programmes. This creates an ethical tension that directly contradicts the core humanitarian principle of ‘leaving no one behind’, as articulated in the United Nations 2030 Agenda for Sustainable Development [10]. This universal value explicitly calls for ending exclusion and reducing the vulnerabilities that leave people behind, including those with disabilities and chronic medical conditions.

This case series reports our experience with eight patients undergoing cochlear implantation under regional anaesthesia during humanitarian missions in Jordan, Kenya, and Senegal. The series demonstrates the feasibility of early activation protocols in resource-variable environments.

This case series has been reported in line with the PROCESS guidelines in the **Online Supplementary Document** [11].

## METHODS

### Study design and setting

This retrospective case series includes eight patients who underwent cochlear implantation under regional anaesthesia during humanitarian surgical missions conducted and organised by a governmental funded institute. Surgeries were performed in Jordan (for Palestinian patients including refugees from Gaza and West Bank), Kenya (for Somali patients, many from refugee camps), and Senegal (for Senegalese patients). Missions were organised in collaboration with local healthcare institutions. Surgeries were performed by four experienced cochlear implant surgeons who perform these surgeries in their daily routine with each having more than 500 successful cochlear implantation experience and selected through national registry that monitors the complication rates. The missions were organised in accordance to the ‘Green Cochlea’ guide [9].

This study was approved by the ethical committee of the University Hospital (Institutional Review Board Number: E-25-10325).

### Patient population

Eight patients (age range 20–66 years; mean age 38.9 years) from three countries (Palestine, Somalia, Senegal) were included.

### Outcome definitions

Success was defined as complete array insertion with no conversion to GA and normal intraoperative telemetry. Early activation was defined as cochlear implant activation within 24 hours of skin closure. Complication was defined as any unexpected event, increasing postoperative discomfort, requiring intervention or prolonging hospitalisation.

### Preoperative assessment and patient selection

Patients underwent comprehensive preoperative evaluation including: otologic examination and audiometry, high-resolution temporal bone CT and MRI, and medical optimisation, including assessment of anaesthesia risk.

Patients were counselled regarding the regional anaesthesia procedure, including expected intraoperative drilling sounds and the need for cooperation.

Only cooperative adult patients were considered for regional anaesthesia cochlear implantation. Each patient underwent comprehensive preoperative counselling explaining the procedure in detail, including expected intraoperative sensations (drilling vibration, irrigation, manipulation), the importance of remaining still, and the ability to communicate with the surgical team throughout the procedure. For patients with profound bilateral hearing loss, communication strategies were established preoperatively, including written instructions, lip-reading, and tactile cues. For the patient with ankylosing spondylitis and fixed neck deformity, regional anaesthesia was specifically chosen to avoid airway management difficulties associated with the need for intubation with general anaesthesia.

### Informed consent and communication strategies

Given the communication barriers inherent in counselling patients with bilateral profound hearing loss, particularly in humanitarian settings where language barrier exists, a multi-modal consent process was employed. The following strategies were used for all patients:

• Written materials: plain-language information sheets were prepared in Arabic (for Palestinian patients), Somali, and French (for Senegalese patients), explaining in simple terms the difference between regional and general anaesthesia, the sensation of drilling and irrigation, the need to remain still, and the option to request the procedure be stopped at any time.

• Communication during counselling: a medical professional interpreter was always the translator if non-Arabic patients were consented. For patients with profound bilateral loss and no usable residual hearing, all counselling was conducted face-to-face with the patient wearing their hearing aids (if available) or with a family member acting as a communication relay. Written questions and answers on a whiteboard were used when lip-reading was not sufficient.

• Third-party verification: where available, local anaesthesiologist (not part of the mission team) were present during consent discussions to ensure patients understood they could refuse without affecting access to other humanitarian services.

• Documentation: all patients provided written informed consent specifically for cochlear implantation under regional anaesthesia, with explicit acknowledgment that conversion to general anaesthesia would be performed if needed. Consent was witnessed by a local healthcare provider fluent in the patient's primary language.

We acknowledge that the inherent power dynamics of humanitarian missions – where patients may feel obligated to accept whatever care is offered – cannot be fully eliminated. However, the presence of local, independent clinicians during consent discussions and the explicit statement that refusal would not affect other care access were intended to mitigate this risk.

### Anaesthesia protocol

All procedures were performed under regional anaesthesia with conscious sedation when needed using a standardised protocol adapted from established techniques for awake otologic surgery. The protocol was designed to maintain patient comfort, cooperation, and spontaneous ventilation throughout the procedure.

The anaesthesia protocol was adapted from established techniques described in the literature.

### Preoperative preparation

#### Fasting and intravenous access

Standard fasting guidelines were observed (six hours for solids, two hours for clear liquids). Peripheral intravenous access was established prior to patient transport to the operating room.

#### Premedication

At the discretion of the anaesthesia provider, optional premedication with midazolam 0.5–1 mg intravenously was administered for anxiolysis, taking care to avoid over-sedation that might impair cooperation.

#### Topical anaesthesia application

To enhance patient comfort during the initial stages of the procedure and minimise discomfort from monitoring electrode placement, a topical anaesthetic strategy was employed. Thirty minutes prior to entering the operating room, the following applications were performed:

• External auditory canal: the external auditory canal was filled with lidocaine/prilocaine 2.5%/2.5% cream. This provides deep dermal anaesthesia of the canal skin, reducing discomfort during manipulation near the canal that happens while thinning the canal for optimum facial recess approach. Care was taken to ensure the cream contacted the entire circumference of the canal skin, by applying it using a suction tip connected to the syringe that helps the cream reach the tympanic membrane directly and leaving no air bubble deep in the canal.

• Facial nerve monitoring electrode sites: a layer of lidocaine/prilocaine 2.5%/2.5% cream was applied over the planned sites for facial nerve monitoring electrode placement (typically the nasolabial fold, lateral canthus, and mentalis region). The cream was then covered with an occlusive plastic wound dressing to enhance transdermal absorption and maximise anaesthetic effect.

• Postauricular area: another layer of cream is applied to the sites that will be injected using needle for local infiltration.

It is important to emphasise that the cream formulation of lidocaine/prilocaine cannot be replaced by a gel formulation for this purpose. Gels demonstrate inferior absorption through keratinized skin compared to creams. The cream formulation, particularly when combined with an occlusive dressing, achieves adequate dermal penetration to anesthetise the cutaneous nerve endings, thereby minimising the discomfort associated with both needle electrode placement and the initial regional anaesthetic infiltration. This technique has been shown to improve patient tolerance of awake procedures involving facial nerve monitoring.

#### Regional anaesthetic dose calculation

Maximum allowable doses were calculated preoperatively for each patient, lidocaine 1% with adrenaline: maximum 7 mg/kg, and bupivacaine 0.25%: maximum 2 mg/kg.

These limits were strictly observed to prevent regional anaesthetic systemic toxicity.

### Intraoperative management

#### Regional anaesthesia infiltration technique

A field block of the postauricular region, along the proposed incision line, was performed using a combination of lidocaine 1% with adrenaline 1:100,000 for rapid onset and haemostasis, and bupivacaine 0.25% for prolonged postoperative analgesia. Another injection was directed anteriorly to the posterior wall of the external auditory canal from the post-auricular area. A direct injection through the canal was done also under the superior and posterior canal skin.

Greater auricular and lesser occipital nerve block was then carried out. The injection site for the nerve block was positioned at the posterior border of the vertical mid-point of sternocleidomastoid muscle (SCM), at the level of second and third cervical vertebrae (C 2-3), with the needle directed superficial to the middle scalene muscle. When available, ultrasound guided injection was performed for increased accuracy [12].

The total volume was titrated to achieve adequate surgical anaesthesia while respecting calculated maximum doses. Careful aspiration was performed before each injection to prevent intravascular administration.

#### Sedation protocol (optional)

Dexmedetomidine, a highly selective alpha-2 adrenergic agonist, was the preferred sedative agent due to its unique properties of maintaining cooperative sedation with minimal respiratory depression and analgesic-sparing effects. A loading dose of 0.5–1 μg/kg was administered intravenously over 10 minutes, with slow administration being essential to prevent hemodynamic instability such as hypotension or bradycardia. This was followed by a maintenance infusion of 0.2–0.7 μg/kg/h titrated to effect, with the infusion rate adjusted to maintain the patient calm and comfortable, breathing spontaneously, in continuous verbal contact and responsive to commands, corresponding to a RASS score of −1, −2 (drowsy, light sedation).

Intraoperative monitoring: standard monitoring included electrocardiography, non-invasive blood pressure, pulse oximetry, respiratory rate with capnography and clinical assessment of sedation and agitation by Richmond Agitation-Sedation Scale (RASS). Facial nerve monitoring was utilised with caution due to patient awake status.

### Surgical technique

All procedures were performed using ‘minimalism’ practice, in which minimal intervention to achieve maximum outcome is intended. [9] This practice utilises a small (approximately 3 cm) incision placed in a non-hair-bearing area, obviating the need for shaving. This minor incision also ensures a tight pocket for the internal receiver-stimulator, thereby reducing the risk of postoperative hematoma and seroma and eliminating the need for postoperative pressure dressing. In this practice the surgeons perform under water skeletonisation of the facial nerve, minimising the need for repeated use of the facial nerve stimulation by improving visualisation, and theoretically decreasing heat-induced facial nerve injury [13].

Consistent with the minimalism philosophy, electrode insertion was performed with particular attention to minimising inner ear trauma. The round window membrane was opened with a fine needle, avoiding drill-based exposure and limiting bony overhang removal to the minimum required for electrode insertion. Following membrane opening, suctioning of the endolymph was minimised to avoid inadvertently provoking vertigo.

Key technical considerations for RA surgery included comfortable positioning with head slightly rotated. Draping arranged to allow facial visibility and ample space above the face to prevent claustrophobia. Furthermore, slow, controlled insertion was performed to avoid any dizziness.

### Early activation

Early activation is a feasible and safe strategy for cochlear implantation fitting that has gained popularity in the latest years [14–16]. Early activation of the patients in this study was arranged intraoperatively or immediate postoperatively in the recovery room.

### Follow-up duration

Patients were followed for six months postoperatively, by the time of writing this manuscript, using remote programming, tele-rehabilitation sessions and tele-clinic for otology follow up.

## RESULTS

The study included eight patients who successfully underwent unilateral cochlear implantation under regional anaesthesia. The mean age was 38.8 years (range = 20–66 years), with a predominance of male patients (seven males, one female). Six cases were carried out in Jordan, one in Kenia and one in Senegal. Patients’ demographics and baseline measures are summarised in **Table 1**.

**Table 1:** Summarised patients’ details*

#	Gender	Age	Nationality	Preop hearing level	Preop WRS	Aetiology	Reason for RA	Operative time
1	M	29	Palestinian	Bilateral profound SNHL	16% Bilateral	Congenital	Optional	45 min
2	M	48	Palestinian	Left ear profound SNHL, right ear sever SNHL	Left 12%, right 60%	Otosclerosis	Optional	85 min
3	M	35	Palestinian	Bilateral profound SNHL	0% Bilateral	Congenital	Optional	35 min
4	M	20	Palestinian	Left profound SNHL, right normal	0% Left side	Head trauma	Optional	50 min
5	F	37	Palestinian	Left ear profound SNHL, right ear moderate SNHL	Left 24%, right 80%	Mondini	Optional	75 min
6	M	66	Palestinian	Bilateral profound SNHL	0% Bilateral	Congenital	Cardiac Disease with High Anesthesia Risk	40 min
7	M	24	Somali	Bilateral sever to profound SNHL	52% Bilateral	Congenital	Optional	30 min
8	M	52	Senegal	Bilateral profound SNHL	0% Bilateral	Usher syndrome	Failed Intubation due to Ankylosing Spondylitis	110 min

*Palestinian patients were operated in Jordan, while Somali patients were operated in Kenya.

Mean operative time (skin-to-skin) was 58.7 minutes (range = 30–110 minutes). Operative times varied substantially by case complexity. The shortest case (30 minutes) was a straightforward congenital profound loss in a young, cooperative patient with normal anatomy (Case 7). The longest case (110 minutes) was the patient with ankylosing spondylitis and fixed neck deformity (Case 8), which required extended time for patient positioning (including modified head support without neck extension, bed rotation to optimise surgeon visualisation while the surgeon stood), repeated intraoperative communication *via* tactile cues due to the patient's blindness, and cautious drilling given the abnormal cervical alignment limiting head stabilisation. The otosclerosis case (Case 2, 85 minutes) required scala vestibuli cochleostomy due to basal turn ossification. The Mondini/EVA case (Case 5, 75 minutes) required meticulous round window identification and minimised suctioning to avoid vertigo. The remaining cases (Cases 1,3,4,6) ranged from 35–50 minutes.

### Sedation and intraoperative patient experience

Of the eight patients, six declined sedation entirely and underwent the procedure using regional anaesthesia alone, reporting only awareness of drilling vibration without significant anxiety.

The two patients (Case 2 and Case 6) who received dexmedetomidine, the mean loading dose was 0.7 μg/kg (range 0.5-1.0) and mean maintenance infusion was 0.4 μg/kg/h (range 0.2-0.7). Target RASS score of −1 to −2 was achieved in both. One patient (Case 2, otosclerosis) required supplemental midazolam 1 mg intravenously during bony canal thinning due to discomfort despite regional anaesthesia. This was the same patient who subsequently developed transient postoperative facial weakness (described below). No other patients required midazolam or additional analgesics beyond the regional block.

No cases required intraoperative conversion to general anaesthesia. The use of regional aanesthesia was associated with a subjective minor prolongation of surgical time noticed by the surgeons, primarily due to temporary interruptions for patient head movements and the occasional need for supplemental analgesia, both of which are not encountered under general anaesthesia. On the other hand, the overall operative room utilisation is decreased due to the omission of the time needed for general anaesthesia including pre-oxygenation, induction, intubation, extubation and emergence.

Electrodes used included MED-EL Flex26 (n = 7) and Form 24 (n = 1). The choice of electrode was based on individual cochlear anatomy using preoperative surgical planning software Otoplan. The electrode insertion was well-tolerated by all patients. Notably, no dizziness or vertigo was reported during electrode insertion, suggesting that atraumatic surgical technique. Complete electrode insertion was achieved in all eight patients. Post-insertion device integrity testing revealed normal impedance values and intact impedance matrices across all electrodes. Furthermore, electrically evoked compound action potential (ECAP) responses were successfully recorded from all contacts, confirming optimal electrode-nerve interface and device functionality.

Five patients underwent successful early activation intraoperatively and three patients underwent immediate postoperatively in the recovery room. At initial activation: all devices functioned normally with appropriate impedance telemetry, patients demonstrated auditory awareness at initial stimulation, and no adverse events (pain, vertigo, facial stimulation) occurred during initial fitting.

One patient had intraoperative pain on thinning the posterior bony canal. The surgeon used 10% lidocaine soaked cotton ball over the canal. This patient developed postoperative grade two facial nerve weakness, that improved completely six hours postoperatively. No other intraoperative or immediate postoperative complications occurred. Specifically, no wound complications, hematoma or seroma, no facial nerve injuries, no vertigo or significant dizziness requiring prolonged recovery, no extracochlear insertion and no device malfunctions.

All patients were discharged on the next postoperative day, consistent with humanitarian mission protocols. All patients affirmed that they would recommend cochlear implantation under regional anaesthesia to others. When asked whether they would have preferred to delay activation beyond one week postoperatively, all responded negatively, indicating satisfaction with the early activation protocol.

### Specific case highlights

Case 2 (48-year-old female, otosclerosis): this patient presented with profound hearing loss on the left and severe loss on the right, with imaging findings consistent with otosclerosis and basal turn ossification of scala tympani. Cochlear implantation under LA was well tolerated despite the potential for increased drilling time due to otosclerotic bone. Scala vestibuli cochleostomy was used for insertion.

Case 5 (37-year-old female, Mondini, enlarged vestibular aqueduct): this patient had bilateral dilated vestibules and EVA, with profound loss on the left and moderate loss on the right. Consistent with the Minimalism approach, suctioning of perilymph during round window opening was strictly minimied. This precaution allowed the patient to tolerate the manoeuvre without vertigo or dizziness.

Case 8 (52-year-old male, blindness, ankylosing spondylitis): this patient presented with bilateral profound SNHL, complete blindness, and advanced ankylosing spondylitis resulting in fixed cervical spine deformity with limited neck extension. General anaesthesia was tried with failed intubation. LA eliminated airway concerns and allowed safe positioning despite the fixed neck deformity. The surgeon performed the surgery standing, the patient’s head was supported with a pillow, and the bed was rotated away from the surgeon to help proper visualisation. Communication during surgery was maintained through tactile cues. The procedure was completed without incident. In one case who had single sided deafness the communication was verbal and the patient reported no discomfort regarding the noise of the drill.

## DISCUSSION

This case series demonstrates that cochlear implantation under regional anaesthesia is feasible and safe in humanitarian mission contexts. Our series contributes several novel observations. First, it extends the applicability of LA implantation to humanitarian missions, where resource constraints and patient volume create distinct pressures. Second, it demonstrates success across diverse aetiologies including normal anatomy, otosclerosis, and EVA. Third, it includes medically complex patients (cardiac disease, ankylosing spondylitis with airway concerns) who benefited from avoidance of general anaesthesia.

The 52-year-old Senegalese patient with ankylosing spondylitis and fixed neck deformity exemplifies the value of LA in complex patients. Airway management in advanced ankylosing spondylitis is extremely difficult, with risks of cervical spine injury and inability to intubate. LA completely circumvented these concerns while still allowing successful implantation – a finding consistent with the principle that LA should be considered when GA poses disproportionate risk.

Humanitarian surgical missions face unique operational constraints: limited operating room time, high patient volumes, variable anaesthetic support, and patients who have travelled long distances with limited follow-up capacity. LA cochlear implantation addresses several of these challenges.

Reduced anaesthetic requirements: LA cases require less intensive anaesthetic support and monitoring, conserving resources for other complex cases. This is particularly valuable when anaesthesia providers are in limited supply.

Leave no one behind: During the humanitarian cochlear implant missions the team has limited time and resources. Patients with complex medical conditions and comorbidities pose a particular difficulty for the anaesthesia team and may need extensive preoperative preparation and postoperative monitoring for their medical conditions including postoperative intensive care unit. These parallel-specialised care might not be easy to arrange in humanitarian setting, resulting in inability to serve these patients. LA can provide a proper solution in these situations that ensures no one is left behind. This value is the second universal value defined by the 2030 Agenda for United Nations’ Sustainable Development Goals which aims to ‘end exclusion’ and ‘reduce vulnerabilities that leave people behind’ [10].

Faster recovery and discharge: patients undergoing LA typically recover more quickly, with less postoperative nausea, earlier ambulation, and shorter observation requirements. This facilitates earlier discharge and reduces the postoperative care burden on mission staff. Palestinian and Somali patients were able to go back to their countries second day postop.

Early activation feasibility: the rapid recovery associated with LA enables early activation protocols. In our series, patients were activated on the same operative day, a timeline that would be challenging after general anaesthesia with its associated recovery period. For patients who have travelled long distances, early activation ensures they return home with a functioning device rather than waiting weeks for activation locally, which might not be available in areas of low resources where humanitarian missions target.

Additionally, there is emerging evidence that early activation might results in lowered impedances. This is theorised to lower battery consumption which decreases the burden on the patients [17–21].

Cost-effectiveness: while formal cost analysis was beyond our scope, LA implantation reduces costs through reduced operative room utilisation, anaesthetic drugs and requirements, and shorter hospital stays [5]. In humanitarian contexts where the cost saved can support additional patients, these efficiencies are of utmost values.

### Comparing variables in humanitarian settings to high resource hospital settings

The mean operative time of 58.7 minutes observed during our humanitarian missions is highly consistent with standard times reported across high-resource settings. Systematic reviews of cochlear implantations under regional anaesthesia found that operative times generally range from 52 to 86.7 minutes [5,8]. Therefore, the reported 58.7-minute mean operative time is highly typical and represents an efficient standard of care.

A 0% conversion rate from regional to general anaesthesia in our case series demonstrates that the procedure's tolerability is maintained even outside traditional hospital infrastructure. This is comparable to established standards; Walters *et al*. (2022) reported only a single instance required conversion to general anaesthesia [8].

Our observed complication profile – comprising zero major adverse events and one minor complication – mirrors the baseline safety of high-resource centres. The literature establishes that zero major complications is the standard expectation for this modality [8]. Furthermore, transient facial nerve weakness is a known and expected minor event. Our single instance of transient weakness aligns with the 1.5% incidence rate noted by Walters *et al*. (2022) [8], as well as retrospective cohorts noting similar occurrences [22].

The overnight observation and next-day discharge protocol implemented in this series is consistent with standard regional anaesthesia practices for cochlear implantation. Across multiple studies, postoperative length of stay for regional anaesthesia averages between 0 and 1.15 days (Walters *et al.*, 2022). While some established cohorts successfully implement same-day discharge [22], our utilisation of overnight observation reliably facilitates safe, efficient recovery that ensures patient safety before they return to remote or underserved communities.

### Implications for humanitarian cochlear implant programmes

Our experience suggests that LA cochlear implantation with early activation may

be considered a viable strategy for humanitarian missions. Key programmatic considerations include:

Patient selection: ideal candidates are all motivated adults who are able to cooperate. Complex medical comorbidities may actually favour LA if GA poses disproportionate risk.

Team training: surgeons and anaesthesiologists should be experienced in LA techniques and comfortable with awake surgery. The anaesthesia team should be ready for possibility of switching into general anaesthesia. Pre-mission simulation and protocol review are beneficial.

Follow-up session: even with early activation, referral for audiology and auditory-verbal therapy clinic is critical to educate the patients’ regarding the use of the device and counsel the patients regarding the postoperative care. Mission teams should establish postoperative counselling sessions before discharge.

## CONCLUSIONS

This case series suggest that cochlear implantation under regional anaesthesia is feasible, safe, and effective in humanitarian mission settings across diverse patient populations and anatomical variations. The approach offers particular advantages for medically complex patients, including those with airway challenges, and risks of general anaesthesia. All eight patients were successfully implanted without conversion to general anaesthesia and achieved early activation intraoperatively.

As humanitarian cochlear implant programmes expand globally, LA techniques may be considered a valuable tool for increasing access to hearing rehabilitation in resource-variable environments. With appropriate patient selection, team training, and protocol development, this approach can safely extend the benefits of cochlear implantation to underserved populations worldwide.

## Additional material


Online Supplementary Document

## Data Availability

**Data availability:** The data supporting the findings of this study are available upon request from the corresponding author.
